# COVID-19 Transmission among Healthcare Workers at a Quarantine Facility in Thailand: Genomic and Outbreak Investigations

**DOI:** 10.4269/ajtmh.21-0344

**Published:** 2021-06-25

**Authors:** Kamolthip Atsawawaranunt, Theerarat Kochakarn, Amornmas Kongklieng, Pakkaporn Panwijitkul, Rujira Tragoolpua, Kanyarat Jaradilokkul, Prayuth Kaewmalang, Duangkamon Loesbanluechai, Namfon Kotanan, Khajohn Joonlasak, Elizabeth M. Batty, Anek Mungaomklang, Vichan Pawun, Wasun Chantratita, Thanat Chookajorn

**Affiliations:** 1 Institute for Urban Disease Control and Prevention, Department of Disease Control, Ministry of Public Health, Bangkok, Thailand;; 2 COVID-19 Network Investigations Alliance (CONI), Bangkok, Thailand;; 3 Genomics and Evolutionary Medicine Unit, Center of Excellence in Malaria Research, Faculty of Tropical Medicine, Mahidol University, Bangkok, Thailand;; 4 Department of Virology, Armed Forces Research Institute of Medical Sciences, Bangkok, Thailand;; 5 Mahidol-Oxford Tropical Medicine Research Unit, Faculty of Tropical Medicine, Mahidol University, Bangkok, Thailand;; 6 Centre for Tropical Medicine and Global Health, Nuffield Department of Medicine, University of Oxford, Oxford, United Kingdom;; 7 The Office of Disease Prevention and Control 4 Saraburi, Department of Disease Control, Ministry of Public Health, Bangkok, Thailand

## Abstract

During the COVID-19 pandemic, Thailand implemented a quarantine program at approved quarantine facilities for every international traveler. Here, we report an epidemiological and genomic investigation of a COVID-19 cluster consisting of seven healthcare workers (HCWs) at a quarantine facility and its partnered hospital in Thailand. Outbreak investigations were implemented to obtain contact tracing data and to establish chains of transmission. Genomic sequencing of SARS-CoV-2 with samples within the cohort was performed. Investigations of 951 HCWs and staff with quarantined travelers were implemented to determine the chain of transmission. Genomic and outbreak investigations identified the international travelers infected with the B.1.1.31 SARS-CoV-2 lineage as the source of this outbreak. The genomic data and the investigated timeline revealed a putative transmission chain among HCWs, pointing toward the transmission via the use of common living quarters at the investigated quarantine site. The evaluation of this cohort has led to a policy recommendation on quarantine facility management. International travel quarantine is an important strategy to contain importation of COVID-19 cases. However, a quarantine facility is likely to become a potential hotspot, requiring thorough preventive measures. Reducing the exposure risk by providing private living quarters and scheduling clinical duties at a quarantine site separated from the conventional healthcare workforce have been implemented.

## INTRODUCTION

Healthcare workers (HCWs) play a vital role in the frontline response against the COVID-19 pandemic. Healthcare workers worldwide are at risk of becoming infected while performing clinical duties, leading to substantial loss in the medical workforce and even deaths from COVID-19.^1^ Development of control and preventive measures to protect HCWs is critical and has to be strengthened based on new information gained during the ongoing pandemic.

Thailand has suffered from several COVID-19 importation events during the pandemic.^2^ The Thai Ministry of Public Health requires every international traveler to undergo a mandatory 14-day quarantine either at state-run or private alternative state quarantine (ASQ) facilities. An ASQ facility is a hotel accredited by the Thai health authorities and partnered with a hospital to monitor quarantined individuals for signs and symptoms of COVID-19 and to collect nasopharyngeal/oropharyngeal swabs for real-time polymerase chain reaction (RT-PCR) testing twice on Days 0–5 and Days 11–13 after entering the country. Healthcare workers from a partner hospital conduct infection control, symptoms monitoring, and COVID-19 testing for ASQ sites.^3^

In November 2020, a COVID-19 outbreak occurred among HCWs at a hospital in Bangkok associated with two ASQ facilities. Seven HCWs tested positive with SARS-CoV-2. Here, we report an investigation of this cluster to reveal transmission events and areas of improvement for protecting HCWS from COVID-19.

## MATERIALS AND METHODS

### COVID-19 outbreak investigations.

Governmental public health officials from Institute for Urban Disease Control and Prevention were notified of a confirmed COVID-19 case: a HCW stationed at a private hospital and quarantine facilities. Outbreak investigation was conducted by site surveys, interviews, and reviews of records at the quarantine facilities and the hospital. Contact tracing data were analyzed to notify individuals with exposure risk to undergo isolation and testing. Real-time polymerase chain reaction testing was done from nasopharyngeal/oropharyngeal swabs using a COVID-19 Coronavirus Real Time PCR Kit (Jiangsu Bioperfectus Technologies, Taizhou, China) targeting the *ORF1ab* and *N* genes. The high-risk exposure group was tested for IgG and IgM anti–SARS-CoV-2 ELISA (EUROIMMUN, Lübeck, Germany). Environmental swabbing was performed at the suspected quarantine site to identify potential fomites.

### SARS-CoV-2 genomic sequencing.

Real-time polymerase chain reaction multiplex amplicon sequencing with ARTIC priver version 3 was performed using SuperScript IV reverse transcriptase (Invitrogen, Carlsbad, CA) and Platinum SuperFi DNA polymerase (Invitrogen) with a published protocol from the ARTIC network.^4^^,^^5^ The products were purified using Agencourt AMPure XP (Beckman Coulter, Brea, CA) and analyzed with a Fragment Analyzer (Agilent Technologies, Santa Clara, CA) and a Qubit 4 Fluorometer (Thermo Fisher, Waltham, MA). Library preparation was done using the Nextara Flex kit protocol (Illumina, San Diego, CA) according to the manufacturer’s protocol. The library was pooled and run on MiSeq Reagent kit v2 (Illumina) with 250 bp paired-end sequencing.

### Bioinformatic and phylogenetic analyses.

Sequencing reads were analyzed using the COG-UK Nextflow pipeline (https://github.com/connor-lab/ncov2019-artic-nf). Briefly, the reads were trimmed using Trim Galore v0.6.5 to discard sequencing adapters and poor-quality reads (https://github.com/FelixKrueger/TrimGalore). Then, alignment to SARS-CoV-2 reference sequence (MN908947.3) was performed using BWA v0.7.17.^6^ ARTIC primer trimming, variant calling, and consensus sequence generation were performed using iVar v1.2.2.^7^ PANGO lineage was called from consensus sequence using Pangolin v2.1.10 and PangoLEARN database version 2021-01-06 (https://github.com/cov-lineages/pangolin).^8^ SARS-CoV-2 samples in the B.1.1.31 lineage were downloaded from GISAID on January 11, 2021. Sample names, GISAID ID, collection date, location, and submitting laboratory are provided in Supplemental Table 1. Maximum likelihood phylogenetic tree was calculated using IQ-TREE v1.6.12 with fast tree search mode, GTR+G substitution model, and 1,000 bootstrap replicates.^9^ Dendroscope v3.7.2 was used for phylogenetic tree visualization.^10^

## RESULTS

The first case (A1) at the start of this investigation was a female nursing staff member at a private hospital in Bangkok who reported her symptoms and was confirmed to have COVID-19 by laboratory testing. She was assigned to work at two different ASQs (ASQ A and ASQ B) from November 16 to December 2, 2020. On December 3, 2020, case A1 developed fever with myalgia and tested positive for COVID-19 by RT-PCR. Case A2, a nurse at ASQ A who shared an on-call room with case A1, developed fever and sore throat on December 4, 2020 and subsequently tested positive for COVID-19. Contact tracing of high-risk contacts of the first case revealed five more positive cases (cases A3–A7). Cases A1–A5 were nurses on clinical duty at the quarantine facilities. Cases A6 and A7 were HCWs at the partner hospital. Cases A3, A4, and A5 tested positive on December 5, 2020. Case A6 originally tested negative on December 5, 2020. During isolation due to exposure risk, case A6 developed coughing and myalgia and was subsequently confirmed to be positive by RT-PCR on December 8, 2020. Case A7, a roommate of case A6, was quarantined after being notified of contact risks and tested negative on December 8, 2020. Later, case A7 developed a sore throat and was confirmed to be SARS-CoV-2 positive by RT-PCR on December 12, 2020.

Because the hospital was responsible for two quarantine sites, it was necessary to identify the putative origin of this outbreak. After reviewing the record from ASQ A and ASQ B, four infected individuals (Q1–Q4) quarantined at ASQ A fit the timeline as possible sources of the outbreak. Genomic investigations were carried out to trace the transmission chain. Quarantined infected individuals Q1–Q3 were members of the same household traveling from Russia and were quarantined in the same quarters. Individual Q4 was a traveler from the United Kingdom who was quarantined in separate quarters at the same site. Genomic sequencing was performed using the samples from individuals Q1, Q2, and Q4 (the sample from individual Q3 was not sufficient for further analyses). The genetic repertoires of SARS-CoV-2 in individuals Q1 and Q2 were identical and belong to the B.1.1.31 PANGO lineage, which is commonly found in Russia.^8^ This lineage was not a part of the local transmission in Thailand outside this cohort based on routine genomic surveillance.^2^^,^^11^ The virus from individual Q4 belonged to the B.1.177 lineage. The viral genetic repertoires from cases A1, A2, A3, A4, and A6 perfectly matched (100% sequence similarity) with those from individuals Q1 and Q2 (Supplemental Figure 1; the sample list is provided in Supplemental Table 1). The virus from case A7 had one additional mutation, consistent with a secondary transmission event. The sample from case A5 was RT-PCR positive for only one out of two genes (N gene: *Ct 35.91*) with merely 1.92% of the genome covered by a multiplex enrichment method.^2^^,^^12^ Thus, there is a possibility that case A5 could have been a false positive or could have had low levels of fragmented viral RNA recovered several weeks after an acute infection phase.^12^

**Figure 1.**
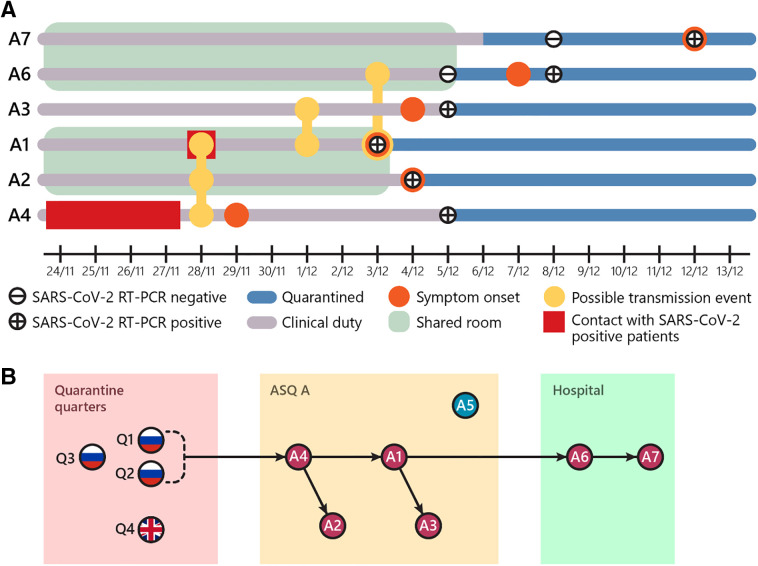
Outbreak timeline and putative transmission events. (**A**) Timeline from healthcare workers (HCWs) infected with SARS-CoV-2. The diagrams represent key events based on outbreak investigations as described in the text. (**B**) Putative transmission chains. The genomic and investigation data were used to determine the transmission chains from infected individuals from Russia and the United Kingdom under travel quarantine. The arrows represent the chains of transmission starting from the Q1-Q2 household to case A4. Based on genome sequencing, infected individual Q4 and case A5 were not included in the transmission chain. The detail of transmission from case A4 to the rest of the HCWs using the investigation data is discussed in the text and presented in the diagram. This figure appears in color at www.ajtmh.org.

Contact tracing and outbreak investigations were conducted based on previous exposures to individuals Q1–Q3 (Figure 1). From the interview and record investigation, case A2 collected nasopharyngeal swabs from individuals Q1–Q3 on November 28, 2020. These three individuals were then confirmed to be positive for SARS-CoV-2 and placed under observation. Personal protective equipment (PPE), including medical protecting coveralls, N95 mask, face shield, hair net, double disposable gloves, and leg covers, were worn during the collection procedure. Case A4 reported contacts with individuals Q1–Q3 in their living quarters during daily temperature and health checks for four consecutive days (November 24–27, 2020). Case A4 gave a history of wearing appropriate PPE during these encounters. Case A4 recalled having upper respiratory symptoms starting on November 29, 2020, but these symptoms were relatively mild. The rest of the hospital staff did not have direct contact with individuals Q1–Q3.

Based on genomic data and the timeline, case A4 was likely to be the first to become infected, probably from the daily health visits with individuals Q1–Q3. It was not possible to sequentially map the chain of transmission based on genomic data alone, and the information from the outbreak investigations was used to track the transmission events. The HCWs shared common living quarters and dining rooms at ASQ A. Transmission from case A4 to cases A1 and A2 could have occurred during mealtime on November 28, 2020. Cases A1 and A2 also shared living quarters at the ASQ facility A. Case A3 had a conversation with A1 on December 1, 2020. Cases A1 and A6 reported having a meal together on December 3, 2020. Case A7 was likely to be infected from A6 because they were roommates. The use of shared facilities was suspected to play a role in this outbreak. In fact, environmental swabs revealed positive RT-PCR results for SARS-CoV-2 from the external bathroom doorknob (RdRP gene cycle threshold [Ct] 28.13, N gene Ct 28.56) inside the living quarters of cases A1 and A2 at ASQ A. Further active case findings at the hospital and the two ASQ facilities with 888 HCWs and 56 hotel staff showed negative RT-PCR results. Serological investigations by ELISA IgM and IgG showed nonreactive results in 29 close contact staff with negative RT-PCR results.

## DISCUSSION

Transmission of COVID-19 originating from a quarantine facility poses a real threat. Hence, strict control measures need to be studied and implemented. The transmission event among HCWs reported here provided insights into potential risks at quarantine facilities worldwide.

Recently, a similar incident occurred at a managed isolation and quarantine facility in Christchurch, New Zealand.^13^ In the Christchurch event, the genomic and epidemiological data suggested that an initial transmission event happened between two quarantined rooms during a brief 50-second gap of door opening between two quarters into the shared corridor. This incident resulted in two known transmission events outside the facility in New Zealand. The information in this report and from the quarantine facility in New Zealand strongly indicate that, due to the highly contagious nature of SARS-CoV-2, implementation of preventive measures has to go beyond a conventional practice between HCWs and infected individuals. Comprehensive planning in facility and staff management is necessary to prevent the spread of COVID-19 from the quarantined facility to the general public.

The routine interaction with infected cases was likely to be the first transmission event here. Quarantined travelers should wear masks at all times when HCWs are to enter their living quarters. Upon contact with quarantined individuals, full PPEs should be worn, ensuring correct donning and doffing of PPEs in a designated area. Waste management of disposal from quarantined living quarters should be handled with care.

In addition, sequential chains of transmission happened because of a series of direct and extensive contacts among HCWs incurred by having them share living quarters and a dining room during clinical rotations. Stricter infection control measures monitored by the Ministry of Public Health need to be implemented for ASQ facilities to avoid non-essential contact with individuals under quarantine. Healthcare workers who are required to stay overnight should be provided with private on-call living quarters. Individuals working in quarantine facilities as well as designated hospitals are at risk of contact with COVID-19–infected travelers and transmitting to other HCWs or patients at the hospital. Scheduling clinical rotations to reduce exposure risks in combination with routine COVID-19 testing would prevent further transmission. The new policy recommendation based on this investigative report has been integrated into the day-to-day activities at the quarantine facility in Thailand (Table 1).

**Table 1:** Protocols and practices for the travel quarantine facilities introduced after the incident.

Before	After
Daily temperature measurement either by quarantined individuals themselves or by healthcare worker dressed in PPE	Minimizing contacts with quarantined individuals by daily self-temperature measurement only with an online record application
No protocol on clinical rotations and living arrangements for HCWs	1. Arrange separate area for work and dinning. HCWs required to stay overnight are provided with private on-call living quarters. 2. Clinical rotation scheduling at quarantine facilities is designed to avoid a direct return to the hospital. This measure is combined with a routine COVID-19 testing in HCWs.

HCW = healthcare worker; PPE = personal protective equipment.
